# A Multimodal Approach to Treatment and Management of Rumination Syndrome in a California Sea Lion (*Zalophus californianus*)

**DOI:** 10.3390/ani15203039

**Published:** 2025-10-20

**Authors:** Amber M. Ramos, Abby McClain, Jennifer M. Dunham, Christian Harris, Jenny Meegan, Barbara K. Linnehan, Kyle P. Ross, Craig Swepston, Mark J. Xitco

**Affiliations:** 1 School of Health in Social Sciences, University of Edinburgh, Edinburgh EH8 9YL, UK; 2 US Navy Marine Mammal Program, San Diego, CA 92152, USA; abby.mcclain@us.navy.mil (A.M.);; 3 The National Marine Mammal Foundation, San Diego, CA 92106, USA

**Keywords:** California sea lion, stereotypy, rumination, megaesophagus, animal behavior, animal welfare

## Abstract

**Simple Summary:**

This case study describes the treatment of a California sea lion with chronic rumination syndrome. The condition involves repetitive regurgitation and re-ingestion of food, which can cause serious medical issues and poor welfare. The animal also presented with an enlarged esophagus and low thyroid function, complicating management. We developed a multimodal plan combining medication, feeding changes, and behavioral strategies. Treatments included thyroid medication, naltrexone to reduce repetitive behaviors, smaller meals delivered in an upright posture, and structured enrichment after feeding. Rumination decreased and eventually ceased. The animal’s health, training participation, and social interactions improved. This case highlights the value of individualized, integrated approaches to complex medical and behavioral conditions in professional care.

**Abstract:**

The management of chronic rumination syndrome in professionally cared-for animals requires a comprehensive and individualized approach. In this case study, a multimodal approach incorporating pharmacological treatment, feeding modifications, and behavioral management was applied to a California sea lion (*Zalophus californianus*) with rumination syndrome, megaesophagus, and hypothyroidism. Behavioral observations were collected through video recordings both prior to and post-intervention. Interventions included oral naltrexone and contingent reinforcement post-feed to provide alternative enrichment activities after feeding sessions to reduce rumination syndrome, as well as levothyroxine for hypothyroidism. Additionally, dietary modifications involved offering smaller food portions, spreading meals across longer time periods, and feeding in a more upright position to facilitate esophageal passage. Results showed a reduction in the frequency of rumination syndrome, with no visible regurgitated material observed five months post-intervention. The sea lion demonstrated improved engagement in training sessions, voluntary husbandry tasks, and open-water activities, as well as improved interactions with conspecifics. The combination of pharmacological, dietary, and behavioral strategies reduced rumination behavior and improved the animal’s overall quality of life, reinforcing the value of individualized care strategies and multimodal treatment plans in addressing complex medical and behavioral comorbidities. These findings show the importance of individualized, multimodal care plans in managing complex behavioral and medical conditions, and they contribute to advancing animal-welfare practices across species.

## 1. Introduction

The care and well-being of animals in zoos and aquariums has become an increasing focus in animal welfare science [1]. Stereotypic behaviors, defined as repetitive, invariant actions without clear function, remain central concerns [2,3,4,5,6]. These may include pacing [5,6], swaying [5,7], self-chewing [8,9], or pica [10,11], and often indicate environmental or psychological stress [4].

Among pinnipeds, chronic rumination syndrome (RS), the repeated regurgitation of undigested food for re-chewing, re-swallowing, or expulsion [12], is of particular concern. RS has been reported in California sea lions (*Zalophus californianus*) [13,14] as well as other species, including gorillas (*Gorilla gorilla*) [15] and bottlenose dolphins (*Tursiops truncatus*) [16]. Health consequences can be severe, including regurgitation of stomach acid [17] weight loss and malnutrition from impaired feeding efficiency [18,19], aspiration pneumonia [20,21], and esophageal disorders such as megaesophagus [18,19,20,22]. Although reports of chronic RS in pinnipeds are limited to isolated case studies and conference proceedings [13,14] this likely reflects underreporting rather than absence. The condition is therefore best regarded as rarely documented but clinically significant, with severe welfare and health implications requiring individualized management.

Megaesophagus, characterized by esophageal enlargement and reduced motility, is linked to diverse etiologies, including hypothyroidism in dogs [23,24,25,26]. Thyroid hormone deficiency may impair neuromuscular function, disrupting peristalsis [23,24]. Although direct associations between RS and megaesophagus are unclear, chronic regurgitation can exacerbate esophageal damage, while hypothyroidism may indirectly contribute through altered gastrointestinal motility [25]. Integrated monitoring of thyroid and esophageal function may be warranted in animals presenting with regurgitation or suspected RS, particularly when accompanied by comorbidities such as hypothyroidism or megaesophagus.

In humans, treatments employ behavioral therapy, pharmacological interventions, and occasionally surgery [27,28,29]. In managed animals, approaches include continuous food access [15,30,31] dietary adjustments [15], and enrichment [16,32]. In California sea lions, effective treatments include frequent training, smaller meals, enrichment, and medications such as digestive enzymes or haloperidol [13,33]. Therefore, management strategies for RS draw on cross-species insights.

Adverse or self-injurious behaviors, such as behavioral regurgitation and reingestion, may also be inadvertently reinforced in zoo settings, where caregiver attention can unintentionally maintain such behaviors [34,35,36]. These behaviors may function as sensory stimulation, escape, attention-seeking, or automatic reinforcement [37,38]. Their multi-factorial nature complicates management in both human and animal populations.

Enhancing pinniped welfare requires individualized, case-based approaches. Such efforts not only benefit the animals but also support conservation messaging and align with the One Welfare framework, which emphasizes connections between animal, human, and environmental well-being [39,40]. Single-subject case studies remain valuable for developing welfare innovations with broader applicability [41].

Here, we report on the multimodal management of RS, megaesophagus, and hypothyroidism in a California sea lion using feeding modifications, behavioral interventions, and pharmacological treatment. We hypothesized that addressing these conditions holistically would mitigate RS symptoms and improve overall well-being.

## 2. Materials and Methods

### 2.1. Focal Animal and Setting

This study was conducted at the Naval Information Warfare Center (NIWC) Pacific in San Diego, California. The Navy’s California sea lions are housed in floating sea pens (9 × 9 m) with adjoining haul-out platforms, where in-pen training typically occurs. These animals have unique opportunities to engage in physically and cognitively stimulating open-ocean tasks, such as diver interdiction and deep-object recovery. The Navy maintains accreditation through the Association for Assessment and Accreditation of Laboratory Animal Care (AAALAC) and complies with the U.S. Public Health Service Policy on the Humane Care and Use of Laboratory Animals and the Animal Welfare Act.

The focal subject was a 17-year-old castrated male California sea lion (CSL) with a documented history of rumination syndrome (RS) first noted when the animal was approximately two years old. Throughout the study, he was housed with conspecifics and participated in two to three structured training sessions daily, with his diet evenly distributed across sessions.

#### 2.1.1. Historical Care

Animals in the U.S. Navy Marine Mammal Program are trained to perform critical missions such as locating sea mines, detecting unauthorized divers, and retrieving objects from the ocean. These tasks require persistence, adaptability, and consistent responsiveness to trainer cues. In this case, RS onset coincided with reduced participation in training, particularly during sessions involving novel tasks. Rumination frequently followed repeated errors, though it was also observed in other contexts. This pattern further limited involvement in open-ocean free-release sessions, which otherwise provide valuable enrichment beyond the home enclosure. During these sessions, rumination was accompanied by social withdrawal, delayed returns, and a lack of responsiveness to trainer cues.

Clinical and behavioral concerns included decreased motivation for fish rewards, low energy relative to age-matched conspecifics, abdominal bloating, and recurrent aspiration pneumonia likely secondary to rumination. Additional stereotypies included atypical gaze, dissociation, and a patterned sequence; head swings, front flipper stands (where the sea lion would raise his hind quarters over his head), and head shaking, typically preceding regurgitation. Similar patterned pre-regurgitation idiosyncrasies have been reported in gorillas in managed care [15,42,43].

Multiple behavioral and pharmacological interventions were attempted with mixed results. Behavioral strategies included ad libitum feeding, satiation, and aversive stimuli (crowding board, loud noise, flipper tapping) to disrupt rumination. Training was also used to encourage retention of regurgitated fish to mitigate weight loss. Pharmacological efforts included selective serotonin reuptake inhibitors (SSRIs), tricyclic antidepressants (TCAs), diazepam, and maropitant citrate. Despite varied approaches, RS persisted with ongoing lethargy, poor training engagement, reduced food motivation, weight fluctuations, and recurrent pneumonia. Treatments were discontinued or modified when ineffective, as determined by veterinary and training staff.

#### 2.1.2. Megaesophagus and Hypothyroidism

Approximately fifteen years after the onset of RS, diagnostic imaging with radiographs and endoscopy revealed esophageal dilation and impaired motility consistent with megaesophagus. Radiographs also showed food material (fish) retained in the esophagus, further indicating reduced esophageal motility. Megaesophagus, characterized by impaired peristalsis and esophageal dilation, is well documented in dogs and cats but less frequently in other mammals [44]. In dogs, acquired forms arise from neuromuscular disease, endocrine disorders (e.g., hypothyroidism, hypoadrenocorticism), esophagitis, or obstruction [45]. In CSLs, *Sarcocystis* infection has been reported as a cause of severe esophageal obstruction and poor prognosis [46].

Given the late onset, this case was presumed to represent acquired megaesophagus. Diagnostic testing ruled out myasthenia gravis, lead toxicity, hypoadrenocorticism, and *Sarcocystis neurona*, but revealed decreased free and total thyroxine (T4) and elevated thyroid stimulating hormone (TSH). Published reference ranges for hypothyroidism in CSLs are lacking, particularly for castrated adult males; therefore, results were compared with values from healthy adult Navy CSLs, confirming abnormal thyroid function. The combination of reduced T4, elevated TSH, lethargy, diminished food motivation, and megaesophagus supported a presumptive diagnosis of hypothyroidism.

### 2.2. Observation and Data Collection

Rumination behavior was monitored using a structured observation protocol. Two closed-circuit television cameras were installed in the enclosure to allow continuous video recording. Focal animal scan sampling was conducted at 20-min intervals to record rumination [47], supplemented by contextual variables (time of day, location, conspecific presence, and concurrent activity).

Observers were trained to ensure accuracy and used playback controls (pause, rewind, zoom) to verify behaviors. Inter-observer reliability, measured during the first three days of analysis, exceeded 98%. Data collection was interrupted when cameras malfunctioned, the CSL was temporarily relocated, or during night shifts without staff. In total, 57 days of usable data were collected across baseline and treatment phases.

### 2.3. Management Plan

Given the coexistence of RS, megaesophagus, and hypothyroidism, a multimodal treatment strategy was designed, incorporating pharmacological therapy, feeding modifications, and behavioral management. Because it was unclear whether RS contributed to, resulted from, or was exacerbated by these comorbidities, a comprehensive approach was necessary. Treatments were introduced sequentially in a multiple-baseline design [48], where each phase served as the baseline for the next. This staggered introduction allowed for direct comparisons across phases and strengthened causal inference by showing that changes in behavior occurred only after each new component was added. The multiple-baseline approach is a rigorous single-subject experimental method because it demonstrates functional relationships without requiring extended untreated baselines or withdrawal of effective interventions, which would not have been ethically appropriate in this case.

#### 2.3.1. Intervention—Levothyroxine

In dogs, hypothyroidism is treated with levothyroxine sodium, typically 0.02 mg/kg orally every 12 h [49,50,51]. Resolution of concurrent megaesophagus following levothyroxine therapy has also been reported [26]. In the absence of CSL-specific dosing guidelines, this animal was started on 0.005 mg/kg (one-quarter of the canine dose) of oral levothyroxine sodium (Thyro-Tabs^®^; Covetrus North America, Dublin, OH, USA) twice daily. Clinical response was monitored through energy levels, food motivation, and rumination frequency, and serum thyroid hormone levels were reassessed six weeks post-initiation.

#### 2.3.2. Intervention–Naltrexone/Contingent Reinforcement Post-Feed (CRPF)

Given the animal’s history of unsuccessful treatment with SSRIs and TCAs, naltrexone (SpecGx LLC, Webster Groves, MO, USA), a non-selective opioid receptor antagonist, was trialed. Naltrexone has reduced stereotypic behaviors in several species including humans [52,53]. A conservative dose of 0.88 mg/kg once daily was used, approximately half the published canine dose [51]. No adverse effects were observed during 24-h post-administration monitoring.

Within days, reductions in post-feed rumination were observed. To reinforce this improvement, a contingent reinforcement post-feed (CRPF) protocol was implemented. After meals, the CSL remained in the dry area of the habitat with a trainer for 60 min. A crowding board, which is routinely present in the sea lion habitat as a standard trainer-safety tool, was briefly positioned under the chest if the animal initiated regurgitation. Crowding boards are commonly used in marine mammal facilities to provide a safe barrier between animals and staff during close interactions. In this context, the board functioned as a physical prompt to maintain an upright posture and prevent expulsion. Its use was limited to the initial days of CRPF and was quickly phased out once posture control was established. CRPF sessions included enrichment (e.g., bubbles, novel objects), rest, non-food-based training with social reinforcement, and voluntary urine collection. Following CRPF, the CSL regained access to water and conspecifics.

#### 2.3.3. Intervention—Feeding Modifications

**Figure 1:**
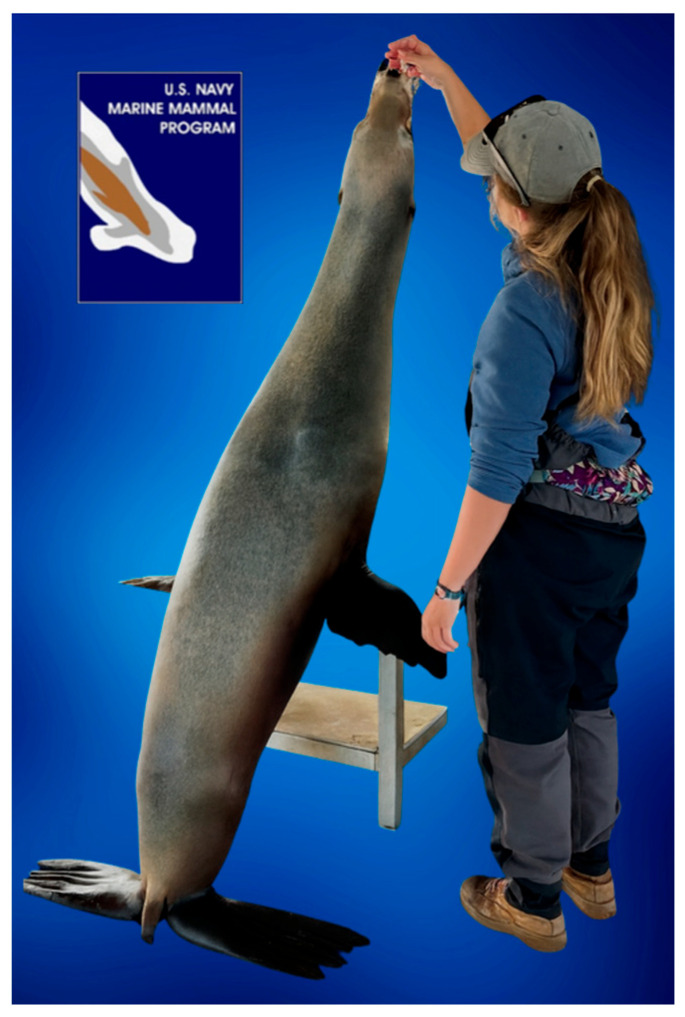
A trainer feeding CSL on a tiered stand, akin to a Bailey Chair.

Feeding was adapted to address megaesophagus and RS. Meals were divided into three daily feedings with fish cut into 2–3 cm pieces to prolong feeding and facilitate esophageal clearance. Sessions were extended to at least 15 min to reduce bolus accumulation. Drawing on canine research indicating that upright feeding aids esophageal transit [54], a modified stand resembling a Bailey chair was constructed. The CSL supported himself with his foreflippers while eating in an upright position, allowing gravity to assist ease of passage through the esophagus (Figure 1).

## 3. Results

**Figure 2:**
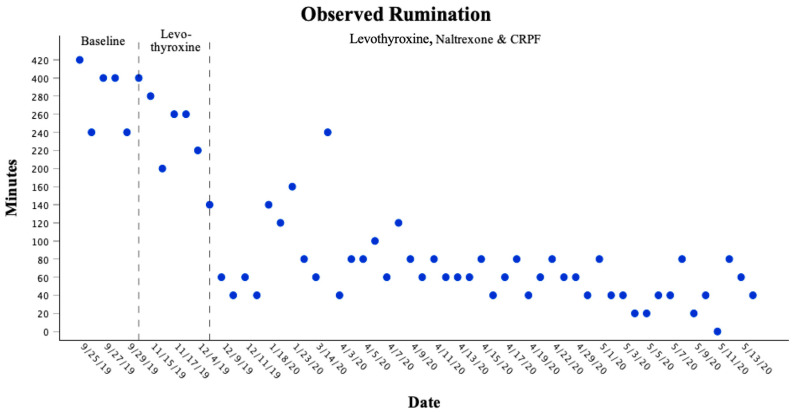
Rumination behavior frequency across treatment phases in 20 min intervals. Rumination remained high during baseline, declined moderately with levothyroxine, and dropped sharply with multimodal treatment.

During baseline observations, before any interventions, the sea lion engaged in rumination behavior for a mean (±SD) of 350 ± 24.8 min/day (*n* = 20 days, range 315–400). This reflected a high and stable rate of rumination under untreated conditions. Following the introduction of levothyroxine therapy, mean daily rumination decreased to 237 ± 27.7 min/day (*n* = 12 days, range 200–276). The reduction was moderate, but variability increased, indicating an inconsistent effect when medication was used alone. When feeding modifications, naltrexone, and contingent reinforcement post-feed (CRPF) were added alongside continued levothyroxine, rumination declined further to a mean of 58 ± 32.0 min/day (*n* = 25 days, range 3–119), representing an 83% reduction compared with baseline. Variability also narrowed during the multimodal phase, reflecting a more stable suppression of the behavior across observation days (Figure 2).

As shown in Figure 2, rumination remained consistently high during baseline, declined moderately with levothyroxine, and then dropped sharply following the multimodal treatment phase, with near-complete elimination of regurgitation events. Notably, although the sea lion continued to display motor patterns historically associated with rumination (e.g., flipper stands, head shaking), the act of regurgitation itself was eliminated. This distinction indicates that while behavioral precursors persisted as remnants, the core pathological behavior was no longer present.

Approximately five months post-intervention, trainers continued to report behaviors historically associated with rumination, but no regurgitated food was observed. To confirm this, cameras were repositioned to optimize visibility, and observers were instructed to specifically document any expulsion of food material. From five to 24 months post-intervention, these stereotypic precursors persisted, but no visible rumination was documented. Since the intervention, no additional episodes of aspiration pneumonia or other illness have occurred. Abdominal distension has been markedly reduced and is now rarely observed.

**Figure 3:**
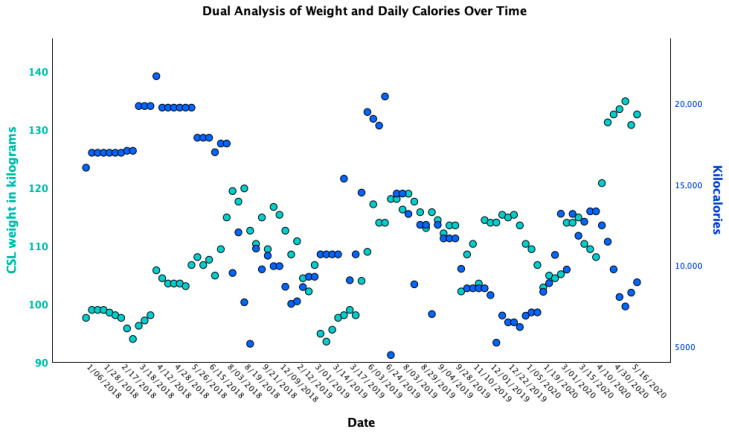
Body weight (kilograms, green) and daily caloric intake (kilocalories, blue) over time. During baseline, high caloric intake was required to maintain a relatively low body weight. During multimodal treatment, caloric intake decreased while body weight increased and stabilized, indicating improved efficiency of caloric utilization.

The animal also demonstrates improved engagement in training, including novel tasks and voluntary husbandry behaviors that had previously been resisted, and has resumed participation in free-release, open-water sessions. Increased affiliative interactions with conspecifics, including initiation of play, have also been observed. The sea lion now maintains a stable body weight with reduced caloric intake requirements (Figure 3). During the baseline phase, high caloric intake was required to maintain a relatively low body weight, whereas during the multimodal phase, caloric intake decreased while body weight increased and stabilized, indicating improved efficiency of caloric utilization.

Follow-up diagnostic imaging via endoscopy and thoracic radiographs revealed qualitatively improved esophageal motility and a reduction in esophageal dilation, confirming a medical improvement associated with the behavioral changes. Representative images are available upon request.

## 4. Discussion

This single-subject case demonstrates that a multimodal strategy including pharmacological treatment (levothyroxine, naltrexone), behavioral management strategies via contingent reinforcement post-feed (CRPF), and dietary modifications, can substantially reduce stereotyped rumination syndrome (RS) in a CSL. Although focused on one animal, the findings emphasize the value of comprehensive, individualized care for complex, co-occurring conditions in professionally cared-for sea lions. Addressing RS alongside hypothyroidism and megaesophagus suggests the promise of integrated approaches for additional species exhibiting similar, maladaptive behaviors.

The modest improvement in RS following initiation of levothyroxine was surprising, but may emphasize the less well-studied relationship between the endocrine system and psychopathology of stereotypic behaviors. A few studies in humans have shown a negative correlation between thyroid hormone levels and self-injurious behaviors where individuals with self-injurious behaviors had lower thyroid hormone levels compared to healthy controls [55,56]. Because naltrexone and CRPF were implemented contemporaneously, isolating their individual effects was not possible; however, their combined introduction was associated with an accelerated decline in RS frequency. This pattern mirrors human literature in which naltrexone is more effective when paired with behavioral interventions for compulsive or addictive conditions, including alcohol use disorder [57,58,59], eating disorders [60], and self-injurious behavior [61].

Beyond endocrine factors, classical neuromodulatory systems may also play a role in the persistence and treatment of rumination. One plausible mechanism is opioid-mediated reinforcement. Rumination and other stereotypic behaviors in humans and animals have been associated with endogenous opioid release, and opioid antagonists such as naltrexone have reduced these behaviors in dogs, primates, and humans [62,63,64]. In this case, naltrexone may have reduced the intrinsic reward value of rumination by blocking opioid receptor activity, thereby weakening the internal reinforcement that maintained the behavior. This pharmacological effect may have allowed contingent reinforcement post-feed (CRPF) and secondary reinforcers to compete more effectively with rumination, producing durable behavioral change.

At the same time, the primary reinforcer in this intervention was food, which may be regulated by different systems, particularly serotonergic pathways. Serotonin has long been implicated in appetite regulation and satiety, with increased serotonergic activity typically associated with reduced feeding drive and enhanced satiety signaling, whereas dysregulation of serotonin function has been linked to compulsive eating and gastrointestinal dysfunction [65,66]. It is possible that the use of food as a reinforcer operated through serotonergic mechanisms, complementing the opioid-antagonist effects of naltrexone [67]. In this way, distinct neurochemical systems may have contributed synergistically: opioid blockade reduced the rewarding properties of rumination itself, while serotonin pathways supported the efficacy of food as a reinforcer in maintaining adaptive behaviors introduced through CRPF.

**Figure 4:**
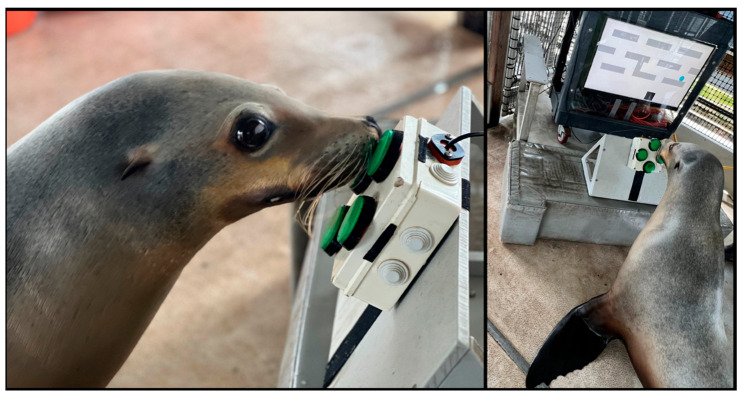
CSL navigating a digital maze using a four-button controller [69].

Animals that voluntarily ruminate may show reduced motivation for primary (diet-based) reinforcers, emphasizing the importance of identifying potent secondary (non-food) reinforcers. During CRPF, without food present, the CSL responded to novel secondary reinforcers (ice, bubbles, toys, music) and opportunities to innovate new behaviors [67,68]. He also engaged with a computerized cognitive enrichment device (Figure 4) without food as reinforcement [69]. Notably, in this individual, task failure had previously been a known antecedent to rumination; however, a subsequent study by Roberts and colleagues [70] observed this individual’s adaptive responses to failure during progressively challenging computerized tasks, deviating from prior behavioral patterns. With increased attention and engagement during trainer-guided sessions, this CSL successfully mastered numerous games within the computerized cognitive device. These included navigating a maze, chasing a moving target (controlled either by software or a human competitor), completing a digital match-to-sample task, and even a menu-based game that allowed him to choose which previously mastered game to play [71].

CRPF was introduced as a new practice after training sessions, replacing the previous routine where the CSL would be sent to the water without further opportunities for interaction or reinforcement from trainers, sometimes for extended periods up to the next day. During CRPF, the animal had the freedom to spend his time as he chose, provided he did not engage in rumination. Allowing animals to have choice and control in their environment and routine is increasingly recognized as central to well-being [72,73,74]. Diminished control can reduce welfare and precipitate maladaptive behavior [75], whereas agency, opportunities to act on the environment, build skills, and tackle manageable challenges, promotes intrinsically motivated behaviors [72]. Contemporary management practices increasingly embed these principles to support behavior-based welfare tailored to individual autonomy [76,77,78,79].

While this effort ultimately proved successful, it was not without its challenges. Achieving consistency in the treatment’s effectiveness required full cooperation from all training staff involved with the animal, necessitating a shift from long-standing practices for an animal that had exhibited rumination behavior for many years. Initially, a considerable amount of time was dedicated to this effort with the addition of CRPF, requiring the full attention of one trainer focused solely on this animal for three hours a day, as well as the time spent recording data from video observations to ensure accurate measures of behavior during baseline and treatment phases. This initiative led to a decrease in veterinary procedures, such as those requiring anesthesia, which were often needed to treat health problems associated with RS in this individual. These complex procedures, which involved several staff members and could last for many hours, became less common as a result.

In humans, rumination syndrome has been reported in association with psychiatric comorbidities such as anxiety, depression, and somatization [80,81,82]. Comparable diagnostic categories cannot be assigned to nonhuman animals, yet certain behavioral observations may reflect functional analogues. In the present case, disengagement after challenging tasks, atypical gaze, and social withdrawal occurred alongside regurgitation episodes. Similar patterns in other species have been interpreted as stress-related coping responses [3,62]. Neurobiological studies of stereotypic behavior suggest potential involvement of endogenous opioid systems and altered reward pathways [63,64]. These parallels remain speculative, and there is currently no evidence directly linking rumination syndrome in pinnipeds to specific mental health disorders. Nevertheless, future research integrating behavioral, physiological, and neurochemical measures may clarify whether mechanisms underlying human psychiatric comorbidities overlap with factors contributing to rumination behaviors in marine mammals.

As the etiology of stereotypies is often unclear, one hypothesis posits that they function as coping strategies during challenging contexts [83]. Modern training and husbandry practices can mitigate risk by shaping achievable steps and minimizing aversive outcomes [84,85]. Successive approximations reduce frustration, aggression, and other adverse reactions [86,87], allowing small wins that buffer stress associated with difficult tasks [88]. Providing choice and control via interactive enrichment (toys, puzzles, novel objects) is associated with enhanced welfare and reduced stereotypy [89]. Incorporating individual preferences into daily routines reflects evolving practice aimed at mitigating stress and promoting well-being [90]. These strategies, necessitating a collaborative effort from veterinary and animal care staff, with considerable upfront investment, reinforce a broader commitment within the animal welfare community [91].

These findings should be interpreted in consideration of the following limitations. Given clear clinical improvement, withdrawing effective components to parse individual effects would have been unethical; thus, relative contributions of each intervention element cannot be determined. Consequently, the generalizability of these results to other animals or to the management of different stereotypic behaviors remains uncertain, yet promising. Broader application across individuals and forms of stereotypy is warranted to evaluate replicability and refine best practices. Such work could inform more widely applicable, welfare-improving protocols.

## 5. Conclusions

In summary, this case highlights the effectiveness of a tailored, multimodal, and individualized strategy in addressing chronic rumination syndrome (RS) in a professionally cared-for California sea lion. By combining pharmacological treatment, feeding modifications, and structured behavioral management, the intervention substantially reduced RS frequency, addressed relevant comorbidities, eliminated visible regurgitation, and improved the animal’s overall functioning. Importantly, although stereotypic precursors to rumination persisted, the absence of regurgitated material for over five months suggests that RS can be managed successfully even in long-standing cases with complex comorbidities. These findings reinforce the importance of individualized, comprehensive strategies that address RS not as an isolated behavior but as a condition shaped by intertwined medical, environmental, and psychological factors, while also suggesting broader applicability in animal-welfare practice.

## Data Availability

No new data were created or analyzed in this study. Data sharing is not applicable to this article.
